# Predicting the 5-Year Risk of Nonalcoholic Fatty Liver Disease Using Machine Learning Models: Prospective Cohort Study

**DOI:** 10.2196/46891

**Published:** 2023-09-12

**Authors:** Guoqing Huang, Qiankai Jin, Yushan Mao

**Affiliations:** 1 Department of Endocrinology The First Affiliated Hospital of Ningbo University Ningbo China; 2 Health Science Center Ningbo University Ningbo China

**Keywords:** nonalcoholic fatty liver disease, machine learning, independent risk factors, prediction model, model, fatty liver, prevention, liver, prognostic, China, development, validation, risk model, clinical applicability

## Abstract

**Background:**

Nonalcoholic fatty liver disease (NAFLD) has emerged as a worldwide public health issue. Identifying and targeting populations at a heightened risk of developing NAFLD over a 5-year period can help reduce and delay adverse hepatic prognostic events.

**Objective:**

This study aimed to investigate the 5-year incidence of NAFLD in the Chinese population. It also aimed to establish and validate a machine learning model for predicting the 5-year NAFLD risk.

**Methods:**

The study population was derived from a 5-year prospective cohort study. A total of 6196 individuals without NAFLD who underwent health checkups in 2010 at Zhenhai Lianhua Hospital in Ningbo, China, were enrolled in this study. Extreme gradient boosting (XGBoost)–recursive feature elimination, combined with the least absolute shrinkage and selection operator (LASSO), was used to screen for characteristic predictors. A total of 6 machine learning models, namely logistic regression, decision tree, support vector machine, random forest, categorical boosting, and XGBoost, were utilized in the construction of a 5-year risk model for NAFLD. Hyperparameter optimization of the predictive model was performed in the training set, and a further evaluation of the model performance was carried out in the internal and external validation sets.

**Results:**

The 5-year incidence of NAFLD was 18.64% (n=1155) in the study population. We screened 11 predictors for risk prediction model construction. After the hyperparameter optimization, CatBoost demonstrated the best prediction performance in the training set, with an area under the receiver operating characteristic (AUROC) curve of 0.810 (95% CI 0.768-0.852). Logistic regression showed the best prediction performance in the internal and external validation sets, with AUROC curves of 0.778 (95% CI 0.759-0.794) and 0.806 (95% CI 0.788-0.821), respectively. The development of web-based calculators has enhanced the clinical feasibility of the risk prediction model.

**Conclusions:**

Developing and validating machine learning models can aid in predicting which populations are at the highest risk of developing NAFLD over a 5-year period, thereby helping delay and reduce the occurrence of adverse liver prognostic events.

## Introduction

### Background

Nonalcoholic fatty liver disease (NAFLD) is a chronic metabolic liver disease closely related to obesity, dyslipidemia, and insulin resistance. It is characterized by excessive fat deposition in hepatocytes, excluding alcohol and other definite liver-damaging factors. In recent years, coinciding with lifestyle and dietary habits changes, the prevalence of NAFLD has gradually increased in several countries [1,2]. Recent studies have shown that the global prevalence of NAFLD is approximately 25%, varying by region and ethnicity [3]. A meta-analysis in China showed a national NAFLD prevalence rate of 29.2% [4]. The rapid increase in the prevalence of NAFLD carries a substantial economic burden and poses a significant threat to people’s lives and overall health [5], which has become a major public health problem.

NAFLD is emerging as one of the most common causes of chronic liver disease and a major cause of liver-related morbidity and mortality worldwide [6,7]. Without timely intervention, NAFLD may progress from simple steatosis to necrotizing inflammation, liver fibrosis, cirrhosis, or even liver cancer [8]. NAFLD is also considered a hepatic manifestation of the metabolic syndrome because of its close association with metabolic disease disorders such as obesity, dyslipidemia, and diabetes mellitus [3,9]. A growing body of research reveals that NAFLD is a multisystem disease that increases the risk of type 2 diabetes, cardiovascular disease, and chronic kidney disease [7]. In addition, studies have shown that obesity, metabolic syndrome, diabetes, and hyperlipidemia are risk factors for NAFLD [2,10]. Early screening for effective interventions can help reduce and delay the occurrence of adverse prognostic events related to NAFLD.

NAFLD has no specific hepatic biochemical abnormalities or clinical symptoms in its early stages, and it is often detected by imaging during health checks or follow-ups of other diseases [9]. Although liver biopsy remains the gold standard for diagnosing NAFLD as an invasive technique, large-scale clinical application is unlikely [11]. In addition, there are no clinically reliable specific markers; therefore, screening for NAFLD is primarily based on liver ultrasound [12]. Mass screening of health screening populations via ultrasound is not only expensive but also consumes a significant amount of medical resources. Therefore, more researchers have begun developing NAFLD risk prediction models using existing clinical data through machine learning and artificial intelligence [13-17]. Risk prediction models for NAFLD are also available and demonstrate good predictive value, but most are built based on retrospective studies. Zhou et al [15] developed a model for predicting NAFLD risk based on children with obesity and demonstrated it by nomograms. Their model had good clinical discrimination, with an area under the receiver operating characteristic (AUROC) curve of 0.821. Liu et al [18] constructed a NAFLD risk prediction model via a machine learning algorithm based on healthy checkup populations, in which extreme gradient boosting (XGboost) showed excellent clinical predictive value, with an AUROC curve of 0.926. In addition, risk models constructed by integrating clinical biochemical and dietary variables also demonstrated better predictive value, with an AUROC curve of 0.843 [13]. Few studies have been reported on 5-year risk prediction models for NAFLD. A 5-year study in a population without obesity showed that the ratio of low-density lipoprotein (LDL) to high-density lipoprotein (HDL) cholesterol was an independent predictor of NAFLD. Its associated hazard ratio was 1.66 (95% CI 1.38-1.99), and it had a *P* trend <.001 and some predictive value, with an AUROC curve of 0.671 [19].

However, there are still limitations to the currently available NAFLD risk prediction models. Most NAFLD risk models have been developed based on cross-sectional studies [13,15,16], meaning that they use postonset experimental data (case controls) to train the model, which inevitably leads to model overfitting. In addition, the level of evidence for cross-sectional studies is relatively low in epidemiologically relevant studies. Notably, external validation is missing in the current NAFLD risk prediction models, which could render their generalization less powerful. NAFLD is a chronic and progressive disease that does not manifest abruptly.

### Objective

Accordingly, the primary objectives of this study were twofold. First, we aimed to examine the 5-year prevalence of NAFLD and identify the associated risk factors in a healthy population in Ningbo, China. Second, we sought to develop and externally validate risk prediction models that can help with evaluating the NAFLD risk over a 5-year period through prospective cohort studies. This approach offers a valuable opportunity for the early prevention and intervention of NAFLD.

## Methods

### Study Population and Design

**Figure 1:**
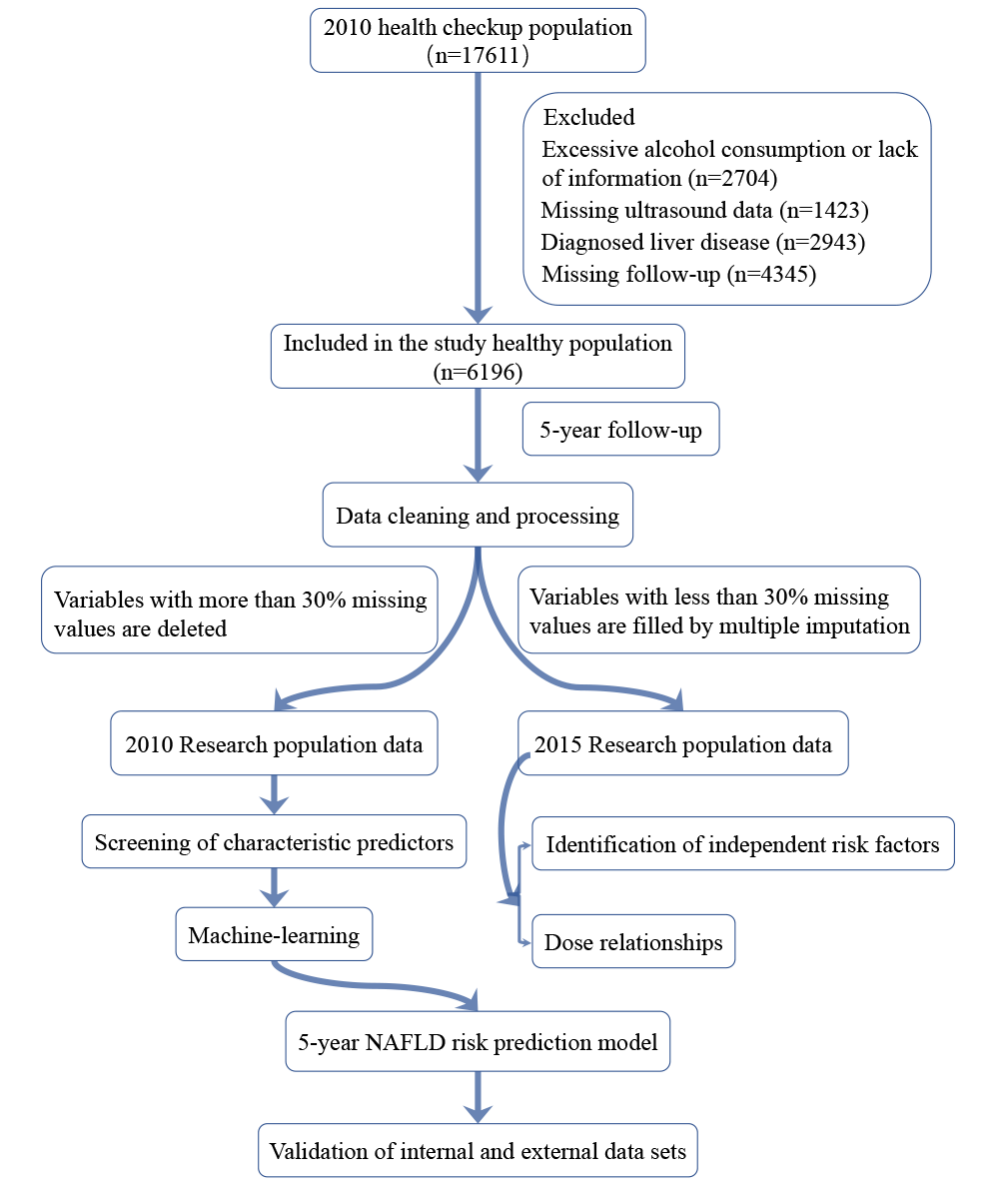
Flowchart of the study. NAFLD: nonalcoholic fatty liver disease.

This study population originated from a long-term follow-up study at Zhenhai Lianhua Hospital in Ningbo, China, which has been reported in the previous literature [20-23]. From the 2010 annual health checkup attendees, we initially gathered a group of 17,611 individuals. Ultimately, 6196 healthy individuals were enrolled after applying the following exclusion criteria: (1) absence of liver ultrasound; (2) diagnosis of liver disease, such as NAFLD, viral hepatitis, and autoimmune hepatitis; (3) alcohol consumption exceeding 140 grams per week for men and 70 grams per week for women; and (4) missing follow-up. Variables with over 30% missingness were removed, and the remaining variables were filled by multiple interpolations (Multimedia Appendix 1) [24]. The study flow is shown in Figure 1.

### Ethics Approval

This study was conducted under the guidance of the STROBE (Strengthening the Reporting of Observational Studies in Epidemiology) statement [25]. The study protocol adhered to the Declaration of Helsinki and was approved by the Ethics Committee of the First Affiliated Hospital of Ningbo University (KY20181209). Informed consent was signed by all participants, and the study data were anonymized.

### Clinical Baseline Data

Height, weight, blood pressure, and waist circumference (WC) measurements were obtained by physical examination. Height and weight measurements required participants to be upright and without shoes and hats. BMI was calculated by dividing weight (kg) by the square of height (m). Before systolic blood pressure (SBP) and diastolic blood pressure (DBP) were measured in the right arm, participants were asked to sit still and rest for 5 minutes. The WC measurements required a horizontal circumference around the abdomen along the midpoint of the line connecting the lower edge of the ipsilateral rib cage and the anterior superior iliac spine. Participants were required to fast for at least 8 hours before venous blood was drawn. Participants’ blood biochemical parameters were measured using either an automated hematology analyzer (Sysmex XT-1800; Sysmex Corp) or an Olympus AU640 automated analyzer (Olympus Optical Corp), following standard protocols [20,23]. Abdominal ultrasound examinations were conducted using a diagnostic ultrasound instrument (Toshiba Medical Systems) evaluated independently by experienced ultrasonographers [26]. NAFLD was diagnosed by the ultrasonographers based on abdominal ultrasound examinations, taking into account the exclusion of excessive alcohol consumption and other etiologies of fatty liver [27].

### Statistical Analyses

The Kolmogorov-Smirnov test was performed to determine whether the samples conformed to a normal distribution. Continuous variables conforming to a normal distribution were described by means and SD, nonnormal continuous variables by median and IQR, and categorical variables by frequency and percentage. Comparisons between continuous variables in the 2 groups were made via an independent sample *t* test or the rank sum test (Mann-Whitney U test) depending on whether they conformed to a normal distribution. Categorical variables were tested by chi-square test, and comparisons were made before and after follow-up via a paired *t* test or paired rank sum test (Mann-Whitney Wilcoxon test). Independent risk factors were identified by multivariate logistic regression analysis. Restricted cubic spline (RCS) was used to assess the dose relationship between the variables and NAFLD. In addition, the sample size of this study complied with the rule of 10 events per variable [28]. All statistic analyses were performed with R (version 4.2.2; R Foundation for Statistical Computing) and Python (version 3.9.0; Python Software Foundation). All tests conducted in this study were 2-tailed, and *P*<.05 was deemed statistically significant.

Participants were randomly assigned to the training and internal validation sets in a 7:3 ratio [29,30]. In addition, to further validate the performance of the prediction model, we used the follow-up population from 2015 to 2020 as an external validation set. We then used extreme gradient boosting (XGBoost)–recursive feature elimination (RFE) combined with the least absolute shrinkage and selection operator (LASSO) to screen the characteristic predictors [31-33]. The Synthetic Minority Oversampling Technique (SMOTE) algorithm was introduced to solve the sample imbalance [24]. A total of 6 machine learning methods, namely logistic regression, decision tree, support vector machine, random forest, categorical boosting, and XGBoost, were used to construct the risk models. The main parameters for the evaluation of the risk prediction model’s effectiveness included the accuracy, precision, *F*-_1_ score, recall, and area under the receiver operating characteristic (AUROC) curve. The calibration curve and Brier score were used to evaluate the degree of model fit.

## Results

### Clinical Baseline Information Before and After Follow-up

This study was derived from a long-term follow-up study, which has been documented in the previous literature. In 2010, 6196 individuals who completed a health checkup (excluding NAFLD) were included in the research. After 5 years of follow-up, we obtained health screening data from this study population again. We statistically described the clinical data of the study before and after the follow-up, and the results are shown in Table 1. During the 5-year follow-up period, a total of 1155 (18.64%) individuals were newly diagnosed with NAFLD, and the incidence was statistically different between the sexes, with an incidence of 941 (81.47%) for male participants and 214 (18.53%) for female participants (*P*<.01). Among the NAFLD population, the lean type (BMI <24 kg/m^2^) was 434 (37.58%), the overweight type (24 kg/m^2^ ≤ BMI < 28 kg/m^2^) was 603 (52.21%), and the obese type (28 kg/m^2^ ≤ BMI) was 118 (10.21%). Based on measurements taken before and after the follow-up, there were slight alterations (*P*<.01) observed within the normal range for BMI, lipids, blood glucose, liver function, and kidney function in the entire population. Compared with the preonset of NAFLD, metabolism-related indicators such as BMI, WC, triglyceride, HDL, apolipoprotein-A1 (Apo-A1), and fasting blood glucose (FBG) increased, and LDL and apolipoprotein-B (Apo-B) decreased. Liver function–related indicators, such as aspartate aminotransferase (AST) and alanine aminotransferase (ALT), were elevated with the onset of NAFLD. In addition, inflammation-related indicators such as white blood cell count (WBC) and neutrophil count were elevated in the postonset NAFLD population.

**Table 1:** Clinical baseline information before and after the follow-up.

Characteristics	NAFLD^a^ population (N=1155)			*P* value	Total population (N=6196)			*P* value
Years	2010	2015			2010	2015		
Male sex, n (%)	941 (81.5)	941 (81.5)	N/A^b^		4107 (66.3)	4107 (66.3)	N/A	
Age (years), mean (SD)	47.47 (10.17)	52.47 (10.17)	N/A		47.97 (10.18)	52.97 (10.18)	N/A	
Body mass index (kg/m^2^), mean (SD)/median (IQR)	24.19 (2.42)	24.84 (2.38)	<.001		22.34 (20.57-24.16)	22.64 (20.76-24.39)	<.001	
Waist circumference (cm), median (IQR)	84 (80-88)	87 (83-92)	<.001		79 (73-84)	81 (75-87)	<.001	
Systolic blood pressure (mmHg), mean (SD)	125.99 (13.83)	129.40 (15.13)	<.001		121.37 (14.89)	123.91 (15.54)	<.001	
Diastolic blood pressure (mmHg), mean (SD)	79.80 (9.86)	79.94 (10.50)	.66		77.34 (10.05)	75.93 (10.71)	<.001	
Heart rate (times/min), mean (SD)	78.75 (12.46)	80.74 (12.01)	<.001		78 (71, 86)	80 (72, 88)	<.001	
White blood cell count (10^9^/L), mean (SD)/median (IQR)	6.32 (1.44)	6.46 (1.43)	<.001		5.70 (4.90-6.70)	5.80 (4.90-6.80)	<.001	
Neutrophil count (10^9^/L), median (IQR)	3.40 (2.80-4)	3.50 (2.90-4.20)	<.001		3.10 (2.50-3.70)	3.20 (2.60-3.90)	<.001	
Eosinophil count (10^9^/L), median (IQR)	0.12 (0.07-0.20)	0.12 (0.08-0.20)	.99		0.11 (0.06-0.18)	0.11 (0.06-0.18)	.64	
Basophil count (10^9^/L), median (IQR)	0.01 (0.01-0.02)	0.01 (0.01-0.02)	<.001		0.01 (0.01-0.02)	0.01 (0.01-0.02)	<.001	
Lymphocyte count (10^9^/L), median (IQR)	2.20 (1.80-2.60)	2.20 (1.90-2.70)	.01		2.10 (1.70-2.50)	2 (1.70-2.40)	.048	
Red blood cell count (10^12^/L), mean (SD)	4.85 (0.42)	4.94 (0.42)	<.001		4.67 (0.44)	4.74 (0.45)	<.001	
Hemoglobin (g/L), median (IQR)	144 (137-151)	150 (142-157)	<.001		140 (128-147)	144 (133-153)	<.001	
Red blood cell distribution width (%), median (IQR)	12.70 (12.30-13.10)	12.50 (12.10-12.90)	<.001		12.70 (12.30-13.10)	12.50 (12.20-12.90)	<.001	
Mean red blood cell volume (fl), median (IQR)	92.20 (89.70-94.80)	91 (88-94)	<.001		92.90 (90.10-95.40)	92 (89-94)	<.001	
Platelet count (10^9^/L), median (IQR)	214 (186-244)	214 (186-248)	.39		211 (182-243)	211 (182-244)	.011	
Platelet distribution width (%), median (IQR)	35.10 (17.40-39.60)	12.10 (11.20-13.30)	<.001		34.80 (16-39.60)	12.10 (11.10-13.30)	<.001	
Mean platelet volume (fl), median (IQR)	10.70 (10.10-11.20)	10.30 (9.80-10.90)	<.001		10.70 (10.20-11.30)	10.30 (9.80-10.90)	<.001	
Alanine aminotransferase (U/L), median (IQR)	19 (14-27)	23 (17-32)	<.001		15 (11-22)	16 (12-23)	<.001	
Aspartate aminotransferase (U/L), median (IQR)	20 (17-25)	21 (18-26)	<.001		19 (16-24)	20 (16-24)	.12	
Total bilirubin (μmol/L), median (IQR)	13 (10-16)	13.40 (10.50-16.90)	<.001		13 (10-16)	13.30 (10.40-17)	<.001	
Direct bilirubin (μmol/L), median (IQR)	4 (3-5)	4.30 (3.60-5.10)	<.001		4 (3-5)	4.10 (3.40-5.10)	<.001	
Direct bilirubin (μmol/L), median (IQR)/mean (SD)	8 (7-11)	9.10 (6.80-11.90)	<.001		9.33 (4.22)	9.91 (4.31)	<.001	
Total protein (g/L), mean (SD)	72.51 (3.45)	73.75 (4.12)	<.001		72.30 (3.59)	73.05 (4.32)	<.001	
Albumin (g/L), mean (SD)/median (IQR)	45.14 (2.14)	46.10 (2.33)	<.001		44.80 (43.30-46.30)	45.80 (44.10-47.40)	<.001	
Globulin (g/L), mean (SD)	27.36 (3.03)	27.64 (3.88)	.003		27.46 (3.03)	27.40 (4.02)	<.001	
Gamma-glutamyl transpeptidase (U/L), median (IQR)	24 (18-33)	29 (20-42)	<.001		18 (14-26)	20 (14-29)	<.001	
Blood urea nitrogen (mmol/L), mean (SD)	4.97 (1.17)	4.83 (1.13)	.003		4.90 (1.17)	4.74 (1.13)	<.001	
Serum creatinine, μmol/L, median (IQR)	67 (58-73)	62 (54-69)	<.001		64 (54-72)	59 (50-67)	<.001	
Uric acid (μmol/L), mean (SD)/median (IQR)	353.06 (76.37)	355.98 (74.01)	.11		316 (260-372)	313 (260-365)	<.001	
Fasting blood glucose (mmol/L), mean (SD)	4.74 (0.44)	5.05 (0.53)	<.001		4.68 (0.43)	4.96 (0.49)	<.001	
Total cholesterol (mmol/L), mean (SD)/median (IQR)	4.91 (0.91)	4.92 (0.87)	.68		4.71 (4.16-5.30)	4.68 (4.16-5.25)	<.001	
Triglyceride (mmol/L), median (IQR)	1.23 (0.91-1.70)	1.54 (1.13-2.09)	<.001		0.96 (0.71-1.34)	1.05 (0.75-1.47)	<.001	
High-density lipoprotein (mmol/L), mean (SD)	1.30 (0.25)	1.41 (0.26)	<.001		1.43 (0.32)	1.52 (0.28)	<.001	
Low-density lipoprotein (mmol/L), mean (SD)	2.85 (0.77)	2.70 (0.68)	<.001		2.77 (0.73)	2.62 (0.65)	<.001	
Apolipoprotein-A1 (g/L), median (IQR)	1.25 (1.12-1.33)	1.36 (1.19-1.57)	<.001		1.28 (1.21-1.39)	1.48 (1.26-1.70)	<.001	
Apolipoprotein-B (g/L), median (IQR)	0.81 (0.66-0.96)	0.77 (0.66-0.86)	<.001		0.73 (0.59-0.87)	0.68 (0.58-0.79)	<.001	
Thyroid-stimulating hormone (mIU/L), median (IQR)	1.50 (1.10-2.10)	1.60 (1.19-2.14)	<.001		1.60 (1.10-2.20)	1.66 (1.21-2.25)	<.001	
Total triiodothyronine (nmol/L), mean (SD)/median (IQR)	1.73 (0.29)	1.72 (0.24)	.70		1.70 (1.50-1.90)	1.68 (1.52-1.83)	.51	
Total tetraiodothyronine (nmol/L), mean (SD)	107.12 (16.87)	109.60 (17.60)	<.001		107.87 (17.44)	110.76 (17.30)	<.001	
Free triiodothyronine (pmol/L), mean (SD)	4.88 (0.61)	4.79 (0.48)	<.001		4.75 (0.62)	4.66 (0.48)	<.001	

^a^NAFLD: nonalcoholic fatty liver disease.

^b^N/A: not applicable.

### Independent Risk Factors

**Figure 2:**
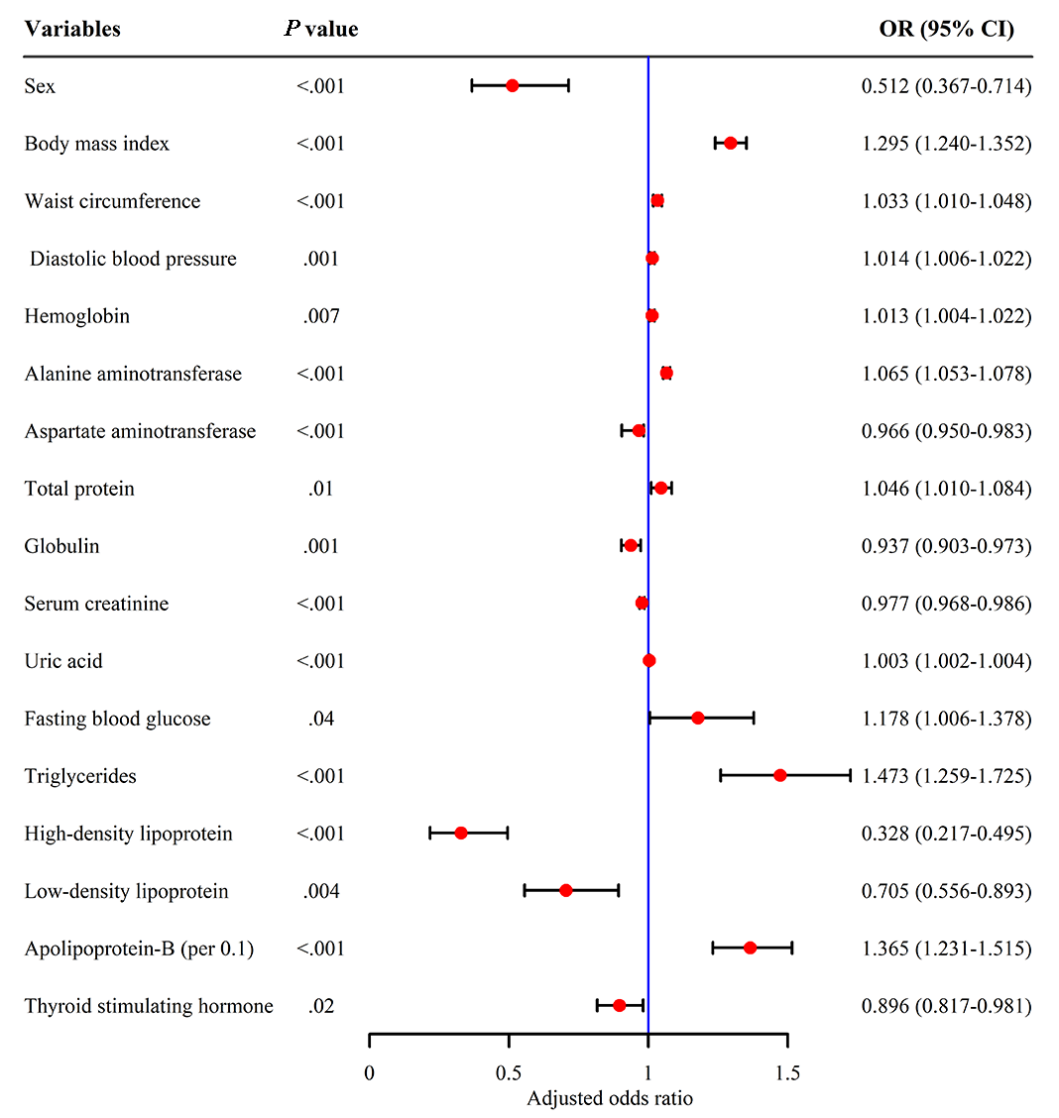
Multivariate logistic regression analysis of nonalcoholic fatty liver disease (NAFLD). OR: odds ratio.

Further, we explored the independent risk of NAFLD based on the 2015 dataset. To this end, 36 potential risk factors associated with NAFLD were screened by univariate analysis (Table 2). Multiple colinearity between variables was tested through variance inflation factor (VIF), which was considered to have severe multiple colinearity between variables when the VIF was greater than 10 (Multimedia Appendix 2). We removed multiple colinear variables by stepwise backward logistic regression, and the final 24 (66.67%) variables were used to screen independent risk factors. Finally, 17 (70.83%) independent risk factors associated with NAFLD, such as BMI, WC, Apo-B, and triglyceride, were identified by multivariate logistic regression (Figure 2).

**Table 2:** Univariate analysis of NAFLD^a^.

Characteristics	Overall (N=6196)	NC^b^ (n=5041)	NAFLD (n=1155)	*P* value
Male sex, n (%)	4107 (66.3)	3166 (62.8)	941 (81.5)	<.001
Age (years), median (IQR)	53 (46-59.25)	53 (46-59)	52 (45-60)	.10
Body mass index (kg/m^2^), mean (SD)	22.69 (2.65)	22.20 (2.45)	24.84 (2.38)	<.001
Waist circumference (cm), median (IQR)	81 (75-87)	80 (74-86)	87 (83-92)	<.001
Systolic blood pressure (mmHg), median (IQR)	123 (113-134)	121 (112-132)	129 (119-138)	<.001
Diastolic blood pressure (mmHg), median (IQR)	75 (68-83)	74 (67-82)	80 (73-87)	<.001
Heart rate (times/min), mean (SD)	80.73 (12.03)	80.73 (12.03)	80.74 (12.01)	.99
White blood cell count (10^9^/L), median (IQR)	5.80 (4.90-6.80)	5.70 (4.80-6.60)	6.30 (5.40-7.30)	<.001
Neutrophil count (10^9^/L), median (IQR)	3.20 (2.60-3.90)	3.10 (2.50-3.80)	3.50 (2.90-4.20)	<.001
Eosinophil count (10^9^/L), median (IQR)	0.11 (0.06-0.18)	0.10 (0.06-0.18)	0.12 (0.08-0.20)	<.001
Basophil count (10^9^/L), median (IQR)	0.01 (0.01-0.02)	0.01 (0.01-0.02)	0.01 (0.01-0.02)	<.001
Lymphocyte count (10^9^/L), median (IQR)	2 (1.70-2.40)	2 (1.70-2.40)	2.20 (1.90-2.70)	<.001
Red blood cell count (10^12^/L), mean (SD)	4.74 (0.45)	4.70 (0.44)	4.94 (0.42)	<.001
Hemoglobin (g/L), median (IQR)	144 (133-153)	143 (131-151)	150 (142-157)	<.001
Red blood cell distribution width (%), median (IQR)	12.50 (12.20-12.90)	12.50 (12.20-13)	12.50 (12.10-12.90)	<.001
Mean red blood cell volume (fl), median (IQR)	92 (89-94)	92 (89-94)	91 (88-94)	<.001
Platelet count (10^9^/L), median (IQR)	211 (182-244)	210 (182-243)	214 (186-248)	.003
Platelet distribution width (%), median (IQR)	12.10 (11.10-13.30)	12.10 (11-13.30)	12.10 (11.20-13.30)	.29
Mean platelet volume (fl), median (IQR)	10.30 (9.80-10.90)	10.30 (9.80-10.90)	10.30 (9.80-10.90)	.49
Alanine aminotransferase (U/L), median (IQR)	16 (12-23)	15 (11-21)	23 (17-32)	<.001
Aspartate aminotransferase (U/L), median (IQR)	20 (16-24)	19 (16-23)	21 (18-26)	<.001
Total bilirubin (μmol/L), median (IQR)	13.30 (10.40-17)	13.30 (10.30-17)	13.40 (10.50-16.90)	.36
Direct bilirubin (μmol/L), median (IQR)	4.10 (3.40-5.10)	4.10 (3.40-5)	4.30 (3.60-5.10)	<.001
Direct bilirubin (μmol/L), median (IQR)	9.10 (6.90-12)	9.10 (6.90-12)	9.10 (6.80-11.90)	.62
Total protein (g/L), mean (SD)	73.05 (4.32)	72.89 (4.35)	73.75 (4.12)	<.001
Albumin (g/L), mean (SD)	45.78 (2.53)	45.70 (2.57)	46.10 (2.33)	<.001
Globulin (g/L), mean (SD)	27.40 (4.02)	27.35 (4.05)	27.64 (3.88)	.03
Gamma-glutamyl transpeptidase (U/L), median (IQR)	20 (14-29)	18 (14-26)	29 (20-42)	<.001
Blood urea nitrogen (mmol/L), median (IQR)	4.64 (3.94-5.42)	4.61 (3.92-5.40)	4.69 (4.02-5.50)	.004
Serum creatinine (μmol/L), mean (SD)	59.13 (11.80)	58.62 (11.81)	61.39 (11.47)	<.001
Uric acid (μmol/L), median (IQR)	313 (260-365)	303 (253-354)	354 (305-405)	<.001
Fasting blood glucose (mmol/L), median (IQR)	4.90 (4.64-5.21)	4.88 (4.63-5.18)	4.98 (4.70-5.32)	<.001
Total cholesterol (mmol/L), median (IQR)	4.68 (4.16-5.25)	4.65 (4.11-5.22)	4.88 (4.36-5.44)	<.001
Triglyceride (mmol/L), median (IQR)	1.05 (0.75-1.47)	0.97 (0.72-1.32)	1.54 (1.13-2.09)	<.001
High-density lipoprotein (mmol/L), median (IQR)	1.50 (1.32-1.70)	1.54 (1.35-1.72)	1.38 (1.22-1.56)	<.001
Low-density lipoprotein (mmol/L), median (IQR)	2.58 (2.18-3)	2.56 (2.16-2.97)	2.66 (2.26-3.10)	<.001
Apolipoprotein-A1 (g/L), median (IQR)	1.48 (1.26-1.70)	1.50 (1.29-1.73)	1.36 (1.19-1.57)	<.001
Apolipoprotein-B (g/L), median (IQR)	0.68 (0.58-0.79)	0.66 (0.57-0.77)	0.77 (0.66-0.86)	<.001
Thyroid-stimulating hormone (mIU/L), median (IQR)	1.66 (1.21-2.25)	1.67 (1.22-2.26)	1.60 (1.19-2.14)	.007
Total triiodothyronine (nmol/L), median (IQR)	1.68 (1.52-1.83)	1.67 (1.52-1.83)	1.71 (1.56-1.87)	<.001
Total tetraiodothyronine (nmol/L), mean (SD)	110.76 (17.30)	111.02 (17.23)	109.60 (17.60)	.012
Free triiodothyronine (pmol/L), median (IQR)	4.63 (4.32-4.96)	4.60 (4.30-4.92)	4.76 (4.46-5.09)	<.001

^a^NAFLD: nonalcoholic fatty liver disease.

^b^NC: normal control.

### Dose Relationship Between BMI, WC, Apo-B, Triglyceride, and NAFLD

**Figure 3:**
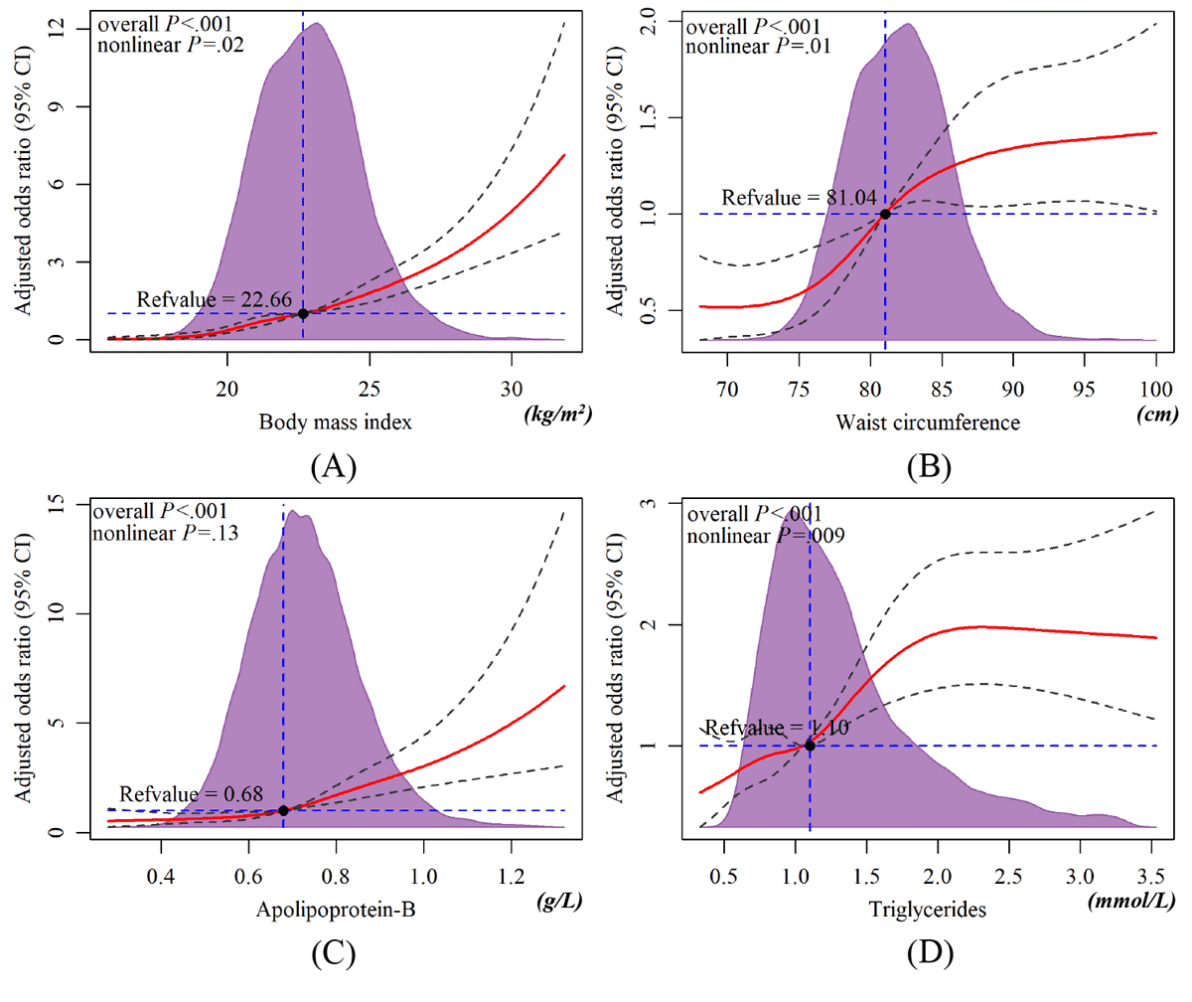
Dose-response relationships between variables and nonalcoholic fatty liver disease (NAFLD). Apo-B: apolipoprotein-B; TG: triglyceride; WC: waist circumference.

Based on the results of multivariate logistic regression, we further explored the relationship between BMI, WC, Apo-B, triglyceride, and NAFLD prevalence. RCS is a common method to explore whether there is a nonlinear association between the independent and dependent variables [34]. In addition, an akaike information criterion was used to screen for the number of knots. We adjusted for confounding factors and performed a nonlinearity test before analyzing the dose-response relationship. From the dose-relationship plot (Figure 3), a nonlinear relationship between BMI, WC, and triglyceride and NAFLD (overall *P*<.05, nonlinear *P*<.05) was found, and the risk of NAFLD increased rapidly when BMI, WC, Apo-B, and triglyceride were greater than 22.66 kg/m^2^, 81.04 cm, 0.68 g/L, and 1.1 mmol/L, respectively. The association between Apo-B and NAFLD was linear (overall *P*<.05, nonlinear *P*>.05), and the risk threshold concentration was 0.69 mmol/L.

### Development and Validation of Predictive Models

**Figure 4:**
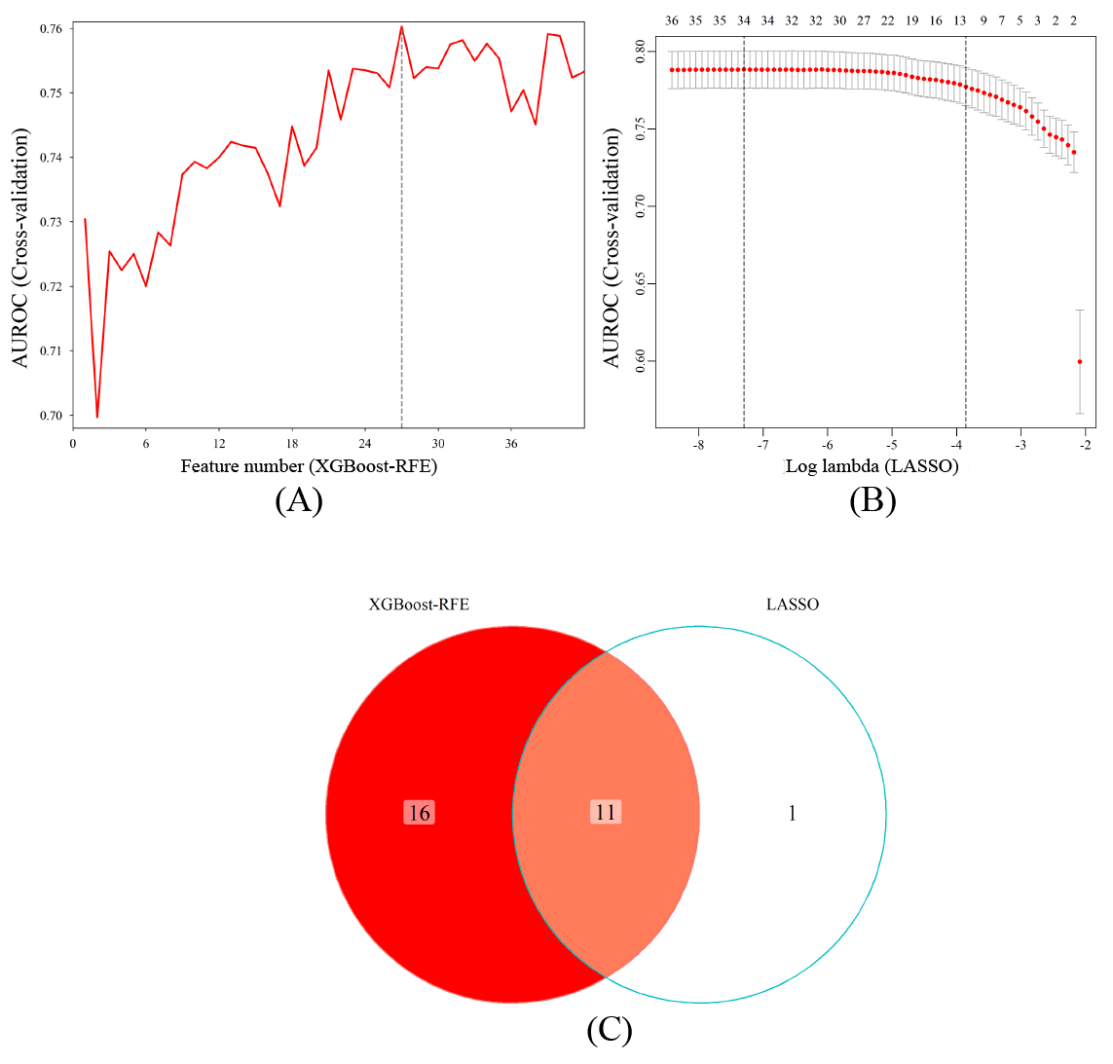
Screening of characteristic predictors. (A) Characteristic variable screening based on XGBoost-RFE. (B) Characteristic variable screening based on LASSO (lambda: 1SE). (C) XGBoost-RFE combined LASSO. AUROC: area under the receiver operating characteristic; LASSO: least absolute shrinkage and selection operator; RFE: recursive feature elimination; SE: standard error; XGBoost: extreme gradient boosting.

XGBoost-RFE enables machine learning algorithms to continuously reduce the number of features and validate the model performance, ultimately achieving the optimal number of features for screening [24]. LASSO is a common method of data dimensionality reduction (without considering multiple colinearities between variables), which compresses the regression coefficients of insignificant variables to 0 by constructing a penalty function, thereby screening the characteristic variables. XGBoost-RFE combined LASSO was used for screening risk predictors of NAFLD (Figure 4). A total of 11 nonzero characteristic variables were screened as predictors for the construction of the 5-year NAFLD risk prediction model (Figure 4C).

**Figure 5:**
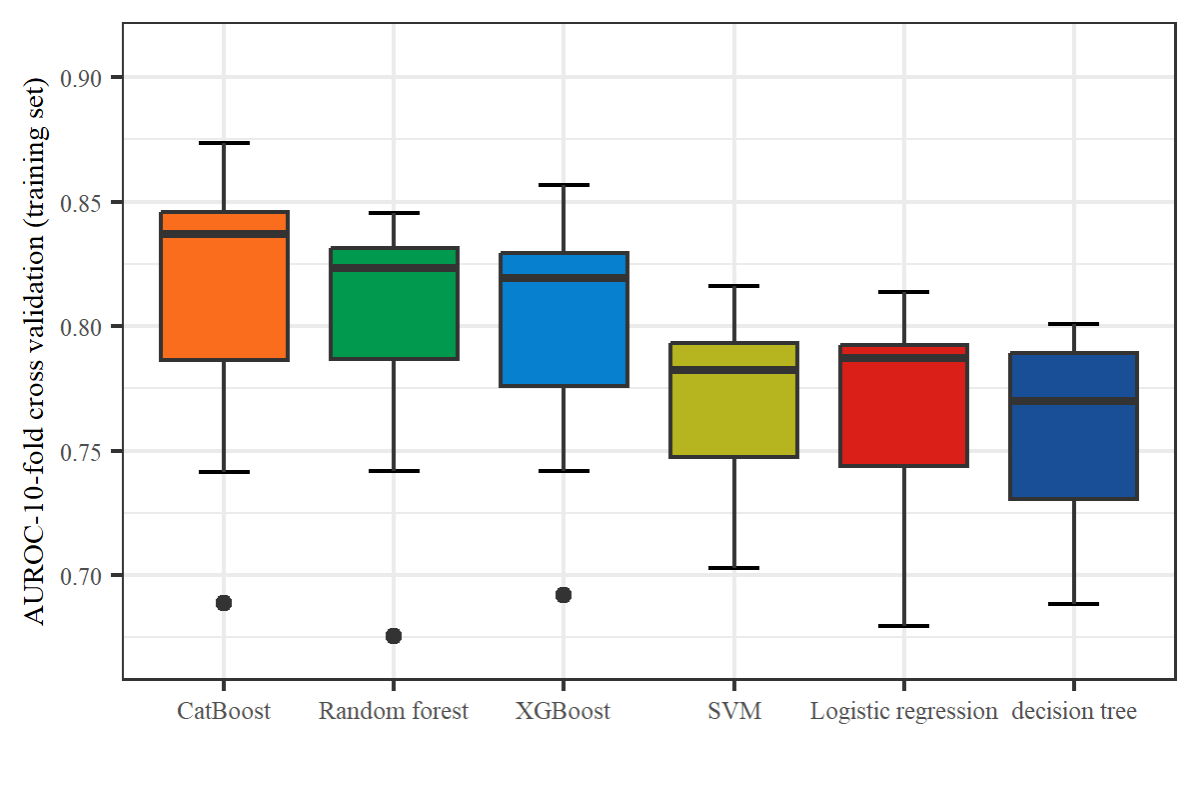
Clinical predictive value of 6 machine learning models (10-fold cross-validation) in the training set. SVM: support vector machine; XGBoost: extreme gradient boosting.

To ensure that each machine model achieved the best performance, we further optimized their hyperparameters (Multimedia Appendix 3). In the training set, 10-fold cross-validation was used to assess the predictive value of the models. As depicted in Figure 5, CatBoost exhibited the highest clinical predictive value, with an AUROC curve of 0.810 (95% CI 0.768-0.852), followed by random forest, with an AUROC curve of 0.800 (95% CI 0.762-0.838).

**Figure 6:**
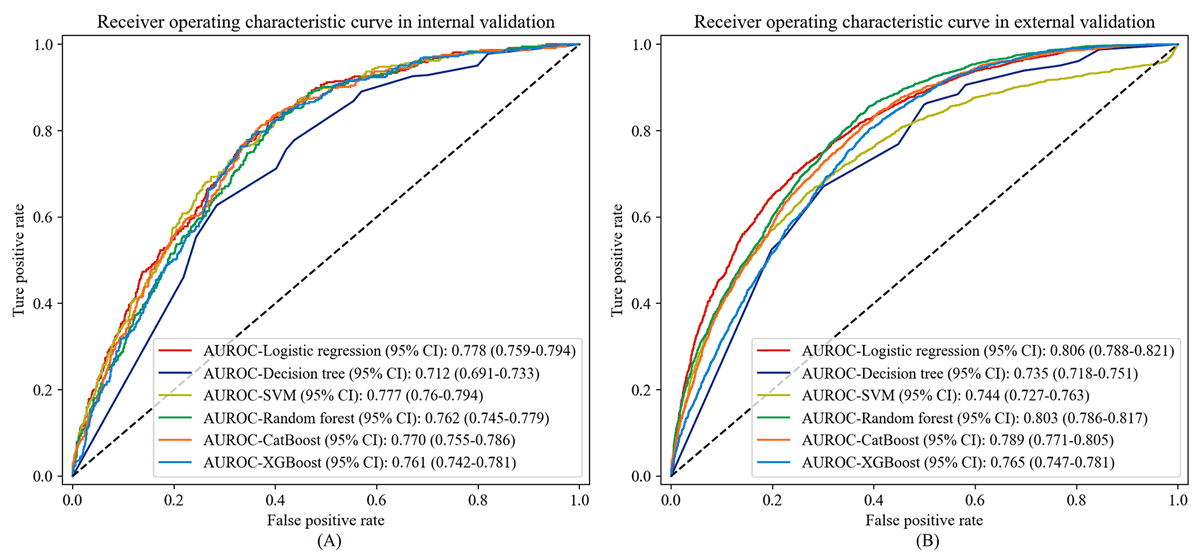
Receiver operating characteristic (ROC) curve of 6 machine learning models in internal and external validation sets. (A) internal validation set; (B) external validation set. AUROC: area under the receiver operating characteristic; XGboost: extreme gradient boosting.

**Figure 7:**
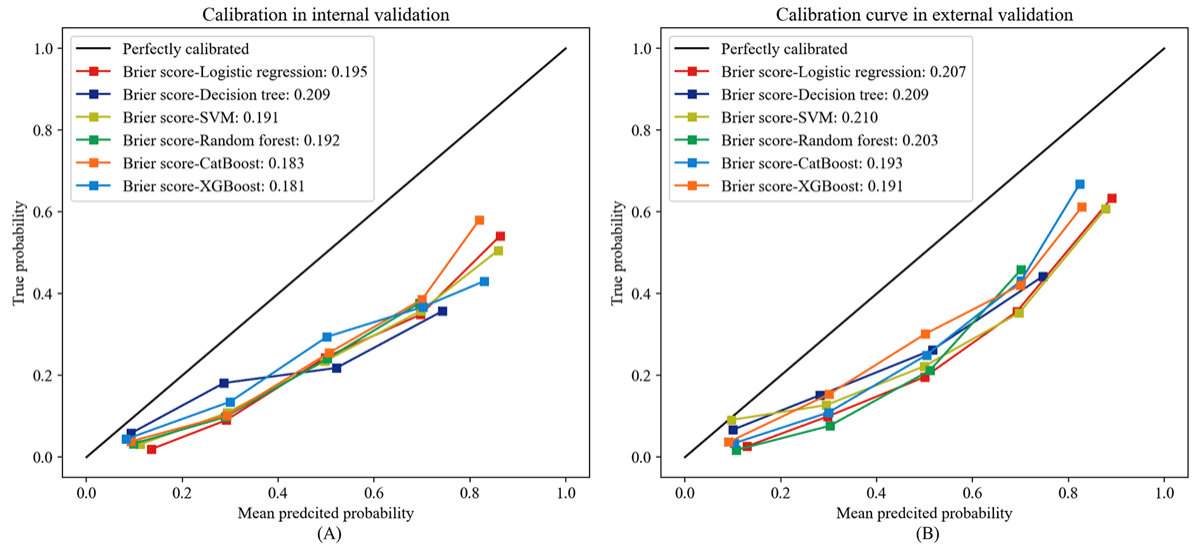
Calibration curve. (A) Internal validation set. (B) External validation set. SVM: support vector machine; XGBoost: extreme gradient boosting.

**Figure 8:**
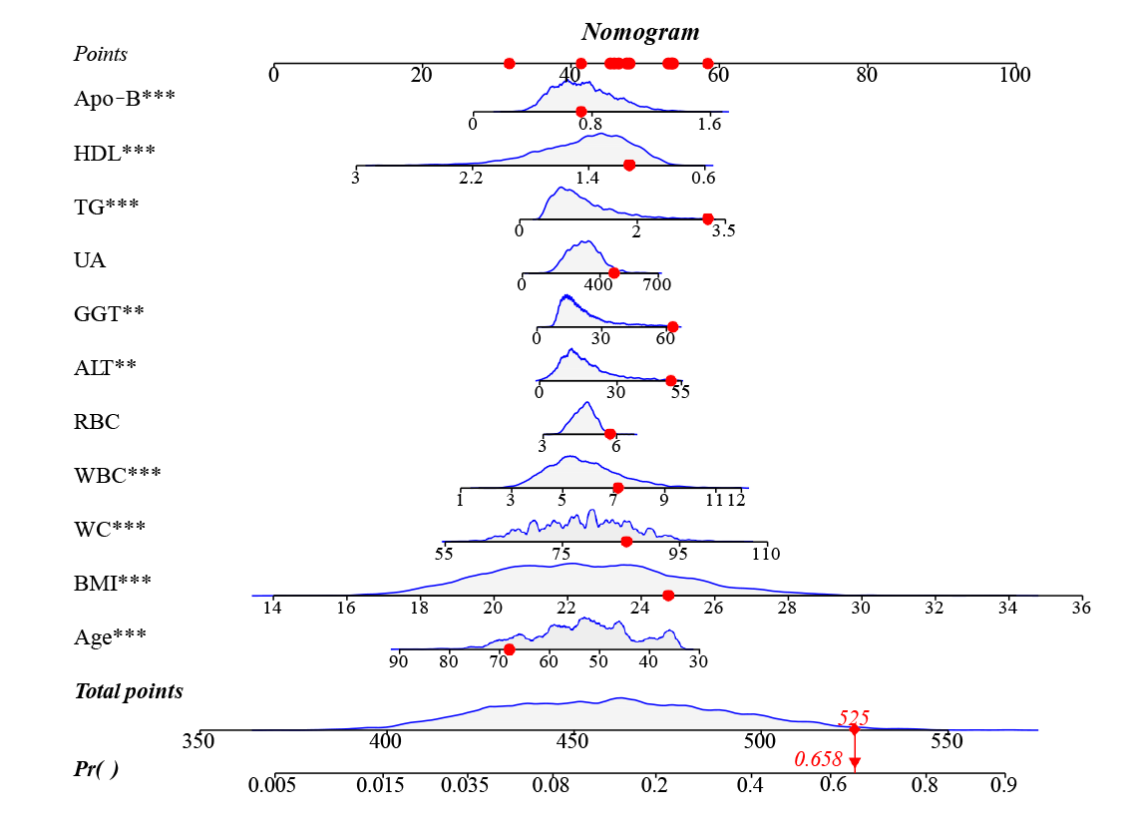
Nomogram for predicting the 5-year risk of developing nonalcoholic fatty liver disease (NAFLD). ALT: alanine aminotransferase; Apo-B: apolipoprotein-B; GGT: gamma-glutamyl transpeptidase; HDL: high-density lipoprotein; RBC: red blood cell count; TG: triglyceride; UA: uric acid; WBC: white blood cell count; WC: waist circumference.

Further, we validated the stability and generalization ability of the 6 predictive models in the internal and external validation sets. Logistic regression models demonstrated the best clinical predictive performance in internal and external validation sets, with AUROC curves of 0.778 (95% CI 0.759-0.794) and 0.806 (95% CI 0.788-0.821), respectively (Figure 6). From Table 3, it is evident that the logistic regression model exhibited favorable performance in terms of accuracy, precision, *F*-_1_ score, and recall in both the internal and external validation sets. In terms of calibration, XGBoost outperformed the other models based on both the internal and external validation sets, with Brier scores of 0.181 and 0.191, respectively (Figure 7). Since the logistic regression model demonstrated the best clinical predictive value in both the internal and external validation sets, we ultimately chose it as the optimal model and demonstrated it with a dynamic nomogram (Figure 4). For example, when a healthy individual is aged 67 years old and has a BMI of 22.86 kg/m^2^, WC of 86 cm, WBC of 5.7 × 10^9^/L, ALT of 51 U/L, gamma-glutamyl transpeptidase (GGT) of 63 U/L, uric acid (UA) of 473 μmol/L, triglyceride of 3.21 mmol/L, HDL of 1.12 mmol/L, and Apo-B of 0.73 g/L, we could infer that their risk of developing NAFLD after 5 years is 65.8% (Figure 8). Thereafter, we developed a web-based calculator to facilitate the prediction model’s application [35].

**Table 3:** Performance parameters of the 6 machine learning prediction models in the internal and external validation sets.

Predictive models			Accuracy		Precision		*F*-_1_ score		Recall
**Internal validation**									
	Logistic regression	0.700		0.637		0.636		0.705	
	Decision tree	0.621		0.599		0.571		0.655	
	Support vector machine	0.688		0.636		0.629		0.707	
	Random forest	0.674		0.628		0.617		0.697	
	CatBoost	0.697		0.632		0.631		0.698	
	XGBoost^a^	0.685		0.633		0.626		0.703	
**External validation**									
	Logistic regression	0.801		0.766		0.648		0.628	
	Decision tree	0.774		0.685		0.654		0.641	
	Support vector machine	0.759		0.879		0.439		0.504	
	Random forest	0.781		0.772		0.565		0.557	
	CatBoost	0.793		0.746		0.633		0.616	
	XGBoost	0.789		0.738		0.623		0.609	

^a^XGboost: extreme gradient boosting.

## Discussion

### Principal Findings

Since 2010, we have conducted a 5-year follow-up study of 6196 participants with health checkups. During the follow-up period, a total of 1155 (18.64%) participants were newly diagnosed with NAFLD. Multivariate logistic regression analysis revealed that 17 variables, including BMI, WC, Apo-B, and triglyceride were independent risk factors for NAFLD. Next, 6 machine learning models were constructed and subjected to hyperparameter optimization. Ultimately, the logistic regression model showcased the best clinical predictive value in the internal and external validation sets, with an AUROC of 0.778 (0.759-0.794) and 0.806 (0.788-0.821), respectively. Additionally, a web-based calculator was developed to assist in the clinical operability of the predictive model.

A recent global meta-analysis showed that the global prevalence of NAFLD after 2016 was 37.8% (between 32.4% and 43.3%), while the annual incidence of new NAFLD was 46.9 (36.4-57.5) per 1000 individuals [36]. The prevalence of NAFLD in China, the largest middle-income country, was 32.9% (between 28.9% and 36.8%) [37], which was lower than the global prevalence. In addition, the latest research indicated that the incidence of NAFLD in China was 5.2% (between 3.9% and 6.5%) [37]. Sun et al [38] showed a 5-year incidence of NAFLD of 14.4% in the population that is not obese. Our study revealed that the 5-year incidence of NAFLD in Zhejiang, China, was 18.64% and the approximate annual incidence was 3.73%, which was lower than the national average. It is known that obesity is strongly associated with many metabolic diseases, including NAFLD. However, NAFLD can also be observed in individuals who are not obese [39]. The prevalence of lean NAFLD varies widely (5% to 45%) due to varying standards of obesity in different countries and regions [40]. In this study’s NAFLD population, the distribution was 37.58% (n=434) lean, 52.21% (n=603) overweight, and 10.21% (n-118) obese.

Multivariate logistic regression analysis showed that BMI, WC, DBP, FBG, triglyceride, HDL, and Apo-B were independent risk factors for NAFLD. All are indicators associated with metabolic syndrome, which suggests that NAFLD is a metabolic-related disease. In addition, recent expert consensus indicates that metabolic dysfunction–associated fatty liver disease (MAFLD) is a better reflection of pathogenesis than NAFLD [41]. Along with the obesity and diabetes epidemic, the disease burden of NAFLD is expected to increase 2- to 3-fold by 2030 in Western countries and Asia [1]. The association of triglyceride, HDL, and Apo-B with NAFLD is consistent with previous reports [42-44]. Further, we found a dose-dependent relationship between BMI, WC, Apo-B, triglyceride, and NAFLD; when they were greater than 22.65 kg/m^2^, 81.04 cm, 1.09 mmol/L, and 0.69 g/L, respectively, an increased risk of NAFLD was observed.

Along with the continuous updating of medical technology, all kinds of medical data are being generated at high speeds [45]. While it is a challenge to mine the data for clinical decisions, in recent years, the emergence and rapid development of machine learning algorithms have facilitated this [17]. Machine learning enables computers to learn from complex clinical big data and solve real-world problems in health care [46].

Our study holds significant clinical significance. This could be one of the first studies to conduct 5-year NAFLD risk prediction based on machine learning methods in a prospective cohort study. Early prevention is better than aggressive treatment. As a chronic progressive liver disease, NAFLD is difficult to reverse once it has occurred. This predictive model can identify individuals at high risk of NAFLD from a healthy population 5 years in advance, providing a significant advantage in the early prevention, diagnosis, and treatment of the disease. Furthermore, the prediction model can not only benefit less medically developed areas but also guide the clinical decisions of physicians, further optimizing health care resources. Finally, convenient web-based calculators provide a medium for the clinical generalization of predictive models.

### Limitations

This may be one of the few tools available for 5-year NAFLD risk prediction in healthy populations. Inevitably, there are some limitations to this study. First, NAFLD was diagnosed by ultrasound methods, and the results may differ somewhat from the actual situation. Second, the prediction model was constructed based on the Chinese population, and whether it applies to other ethnic groups remains to be validated. Third, the collection of clinical data was not comprehensive enough, and potential predictive factors may have been overlooked. In future studies, we will continue to examine and modify the prediction model in clinical practice in collaboration with multiple centers.

### Conclusions

In conclusion, based on a long-term follow-up study in Ningbo, China, we found a 5-year incidence of NAFLD of 18.65% in health checkups. Further, we developed and externally validated a 5-year NAFLD risk prediction model, which is important for the reduction and prevention of adverse liver prognostic events.

## Appendix

Multimedia Appendix 1 Missing values in the study population.

Multimedia Appendix 2 Variance inflation factors for candidate independent risk factors.

Multimedia Appendix 3 Hyperparameter optimization of 6 machine learning models.

## Abbreviations

ALT: alanine aminotransferase
Apo-A1: apolipoprotein-A1
Apo-B: apolipoprotein-B
AST: aspartate aminotransferase
AUROC: area under the receiver operating characteristic
DBP: diastolic blood pressure
FBG: fasting blood glucose
GGT: gamma-glutamyl transpeptidase
HDL: high-density lipoprotein
LASSO: least absolute shrinkage and selection operator
LDL: low-density lipoprotein
MAFLD: metabolic dysfunction–associated fatty liver disease
NAFLD: nonalcoholic fatty liver disease
RBC: red blood cell count
RCS: restricted cubic splines
RFE: recursive feature elimination
SBP: systolic blood pressure
SMOTE: Synthetic Minority Oversampling Technique
UA: uric acid
VIF: variance inflation factor
WBC: white blood cell count
WC: waist circumference
XGBoost: extreme gradient boosting

## Data Availability

The relevant data are available from the corresponding author upon reasonable request.
