# Supplementary figures and images for: Predicting the 5-Year Risk of Nonalcoholic Fatty Liver Disease Using Machine Learning Models: Prospective Cohort Study
Source: J Med Internet Res. 2023 Sep 12;25:e46891. doi: 10.2196/46891 (PMC10523217; doi:10.2196/46891)

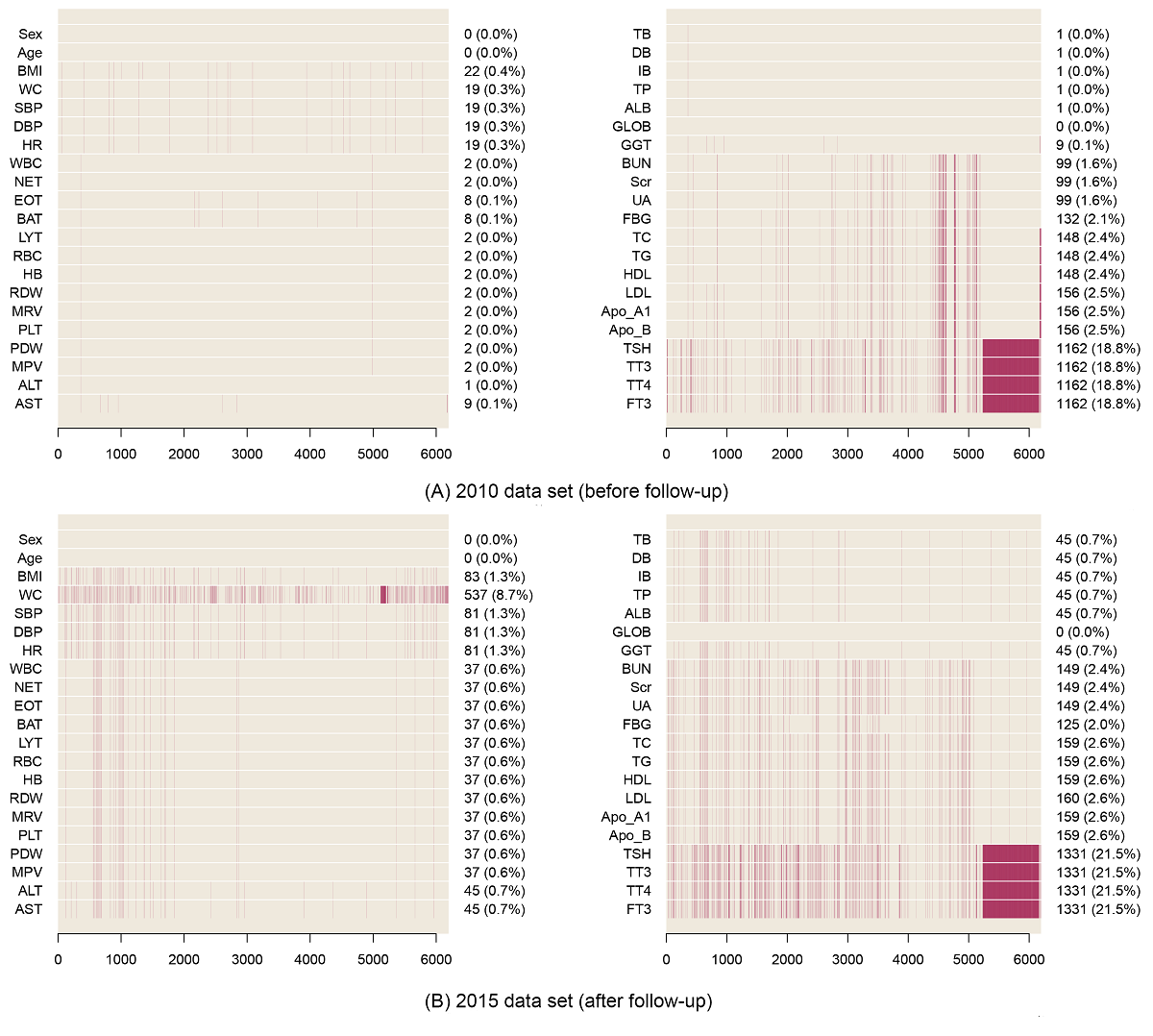

Supplement: Multimedia Appendix 1 [file jmir_v25i1e46891_app1.png]

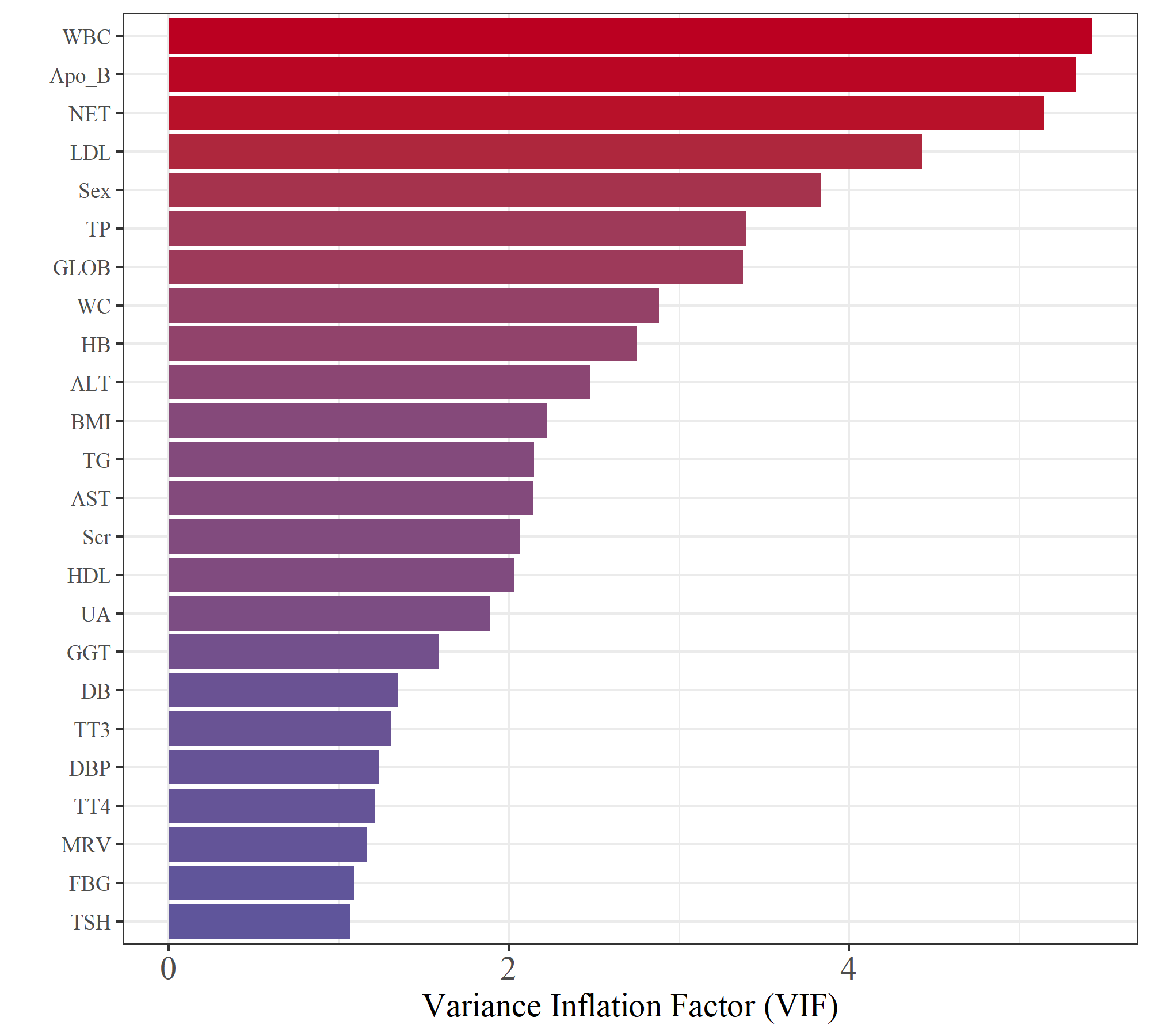

Supplement: Multimedia Appendix 2 [file jmir_v25i1e46891_app2.png]
