# Supplementary material for: Predicting the 5-Year Risk of Nonalcoholic Fatty Liver Disease Using Machine Learning Models: Prospective Cohort Study
Source: J Med Internet Res. 2023 Sep 12;25:e46891. doi: 10.2196/46891 (PMC10523217; doi:10.2196/46891)
Supplement: Multimedia Appendix 3 [file jmir_v25i1e46891_app3.pdf]

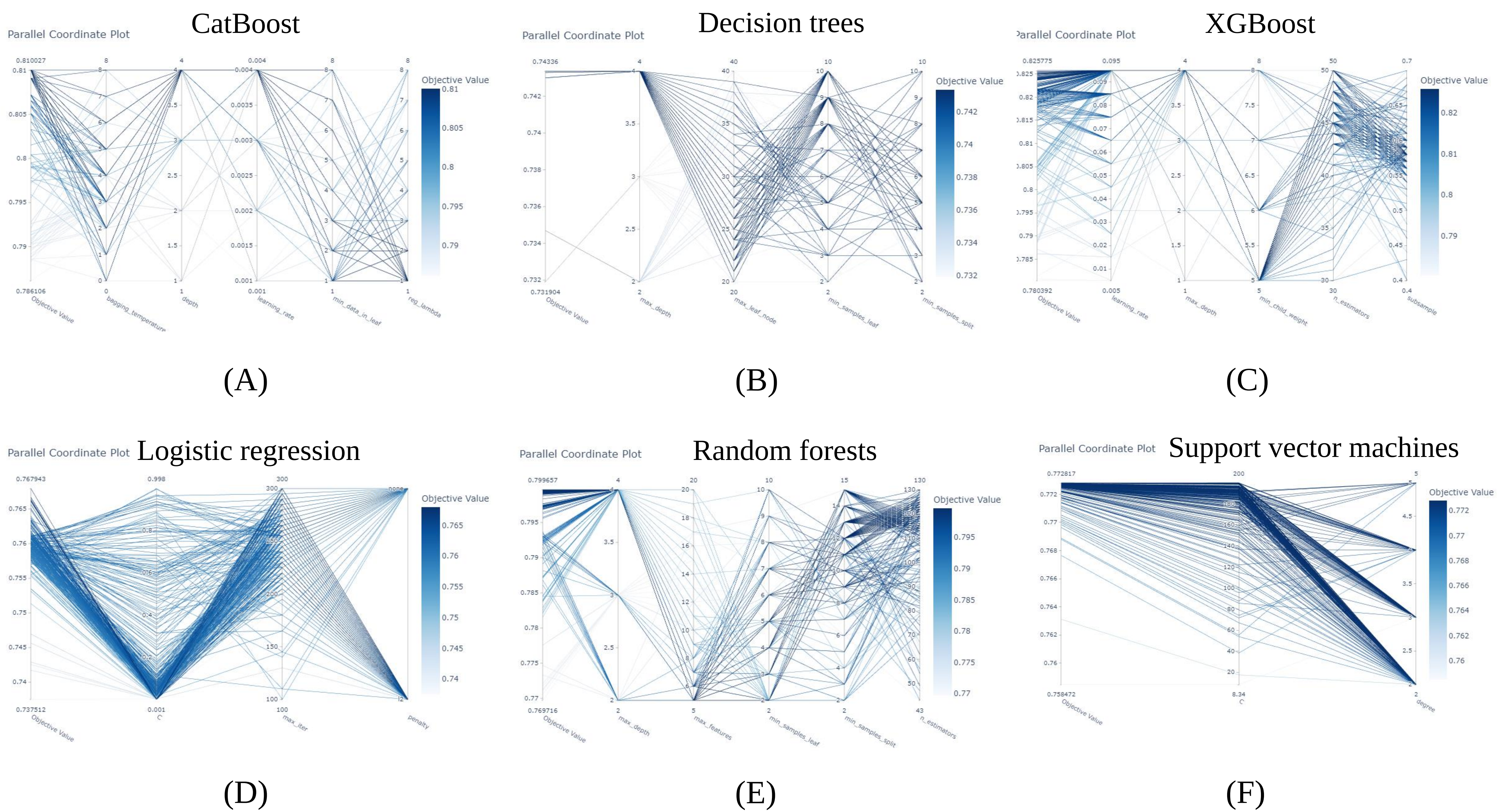

CatBoost
Decision trees
XGBoost
Logistic regression
Random forests
Support vector machines
Parallel Coordinate Plot
Objective Value
bagging_temperature
depth
learning_rate
min_data_in_leaf
reg_lambda
max_depth
max_leaf_node
min_samples_leaf
min_samples_split
min_child_weight
n_estimators
subsample
C
max_iter
penalty
max_features
degree
(A)
(B)
(C)
(D)
(E)
(F)
